# GLP-1R in diabetes mellitus: from basic discovery to therapeutics development

**DOI:** 10.3389/fphar.2025.1610512

**Published:** 2025-05-30

**Authors:** Shujun Li, Nanqu Huang, Mei Wang, Wendi Huang, Yong Luo, Juan Huang

**Affiliations:** ^1^ Key Laboratory of Basic Pharmacology and Joint International Research Laboratory of Ethnomedicine of Ministry of Education, Zunyi Medical University, Zunyi, Guizhou, China; ^2^ National Drug Clinical Trial Institution, Third Affiliated Hospital of Zunyi Medical University (The First People’s Hospital of Zunyi), Zunyi, Guizhou, China; ^3^ Department of Geriatrics, Third Affiliated Hospital of Zunyi Medical University (The First People’s Hospital of Zunyi), Zunyi, Guizhou, China; ^4^ Department of Neurology, Third Affiliated Hospital of Zunyi Medical University (The First People’s Hospital of Zunyi), Zunyi, Guizhou, China; ^5^ Chinese Pharmacological Society-Guizhou Province Joint Laboratory for Pharmacology, Zunyi, Guizhou, China

**Keywords:** glucagon-like peptide-1 receptor, diabetes mellitus, β-cell, insulin secretion, blood glucose

## Abstract

Diabetes mellitus (DM), a metabolic disorder syndrome characterized by persistent hyperglycemia, has a complex pathogenesis. As the number of diabetic patients continues to grow globally, this disease has become a major and growing challenge in global public health. Glucagon-like peptide-1 receptor (GLP-1R) is a G protein-coupled receptor widely expressed on the surface of a wide range of cells in the human body, including pancreatic islet α, β and δ cells, as well as multiple tissues such as the intestines, stomach, lungs, heart, kidneys, and central nervous system. GLP-1R works through the combination of the endogenous ligand Glucagon-like peptide-1 (GLP-1) or exogenous agonists, which activate multiple intracellular signaling pathways that enhance insulin secretion, inhibit glucagon secretion, protect β-cells from apoptosis, delay gastric emptying and increase satiety. This makes GLP-1R a key target for diabetes treatment. This paper reviews the structural and functional characteristics of GLP-1R. Its role in glucose homeostasis and its application in diabetes treatment. It focuses on the mechanism of action of GLP-1R in pancreatic islet α-cells, β-cells and δ-cells, as well as its effects on the central nervous system and gastrointestinal tract. In addition, the article reviews the clinical progress of GLP-1R agonists, including their efficacy, safety and potential in the treatment of diabetes and related complications.

## 1 Introduction

Diabetes mellitus (DM) is a metabolic disease characterized by hyperglycemia (elevated blood glucose), which is caused by a lack of insulin or failure of insulin, resulting in cells being unable to absorb glucose, thus increasing blood glucose levels. Type 2 diabetes mellitus (T2DM) is one of the major subtypes of DM, and its pathogenesis is more complex, involving insulin resistance and insulin secretion dysfunction (Xourafa et al., 2024). The development of insulin resistance is associated with a variety of factors, including obesity, genetic background, and defects in insulin signaling pathways (Saisho, 2015). According to the latest epidemiological data, the number of people with diabetes worldwide has reached 529 million in 2021 and is expected to increase to 1.31 billion by 2050 (Ong et al., 2023).

In recent years, Glucagon-like peptide-1 receptor (GLP-1R) and its agonists have attracted much attention in the field of diabetes treatment. GLP-1R is a core member of the G protein-coupled receptor family and is widely distributed on the surface of many human cells (Zheng et al., 2024). GLP-1R activation has a significant effect on glycemic control in patients with T2DM through a variety of mechanisms, including stimulation of insulin secretion, inhibition of glucagon secretion, protection of β-cells from apoptosis, slowing gastric emptying and increasing satiety (Drucker, 2006). These mechanisms not only help to improve blood glucose levels, but also positively affect the overall metabolic state. In addition, GLP-1R agonists help treat diabetes by protecting the brain and heart, preserving kidney function and aiding weight loss (Abiola et al., 2024). This is especially important for people with diabetes who also have heart issues, kidney problems, or are overweight. In summary, the importance of GLP-1R in the field of diabetes treatment is not only reflected in its precise regulation of blood glucose, but also in its ability to comprehensively manage multiple complications. GLP-1R agonists improve the metabolic status of diabetic patients through a variety of mechanisms, which provides a new strategy and direction for the treatment of T2DM.

## 2 GLP-1R

**FIGURE 1:**
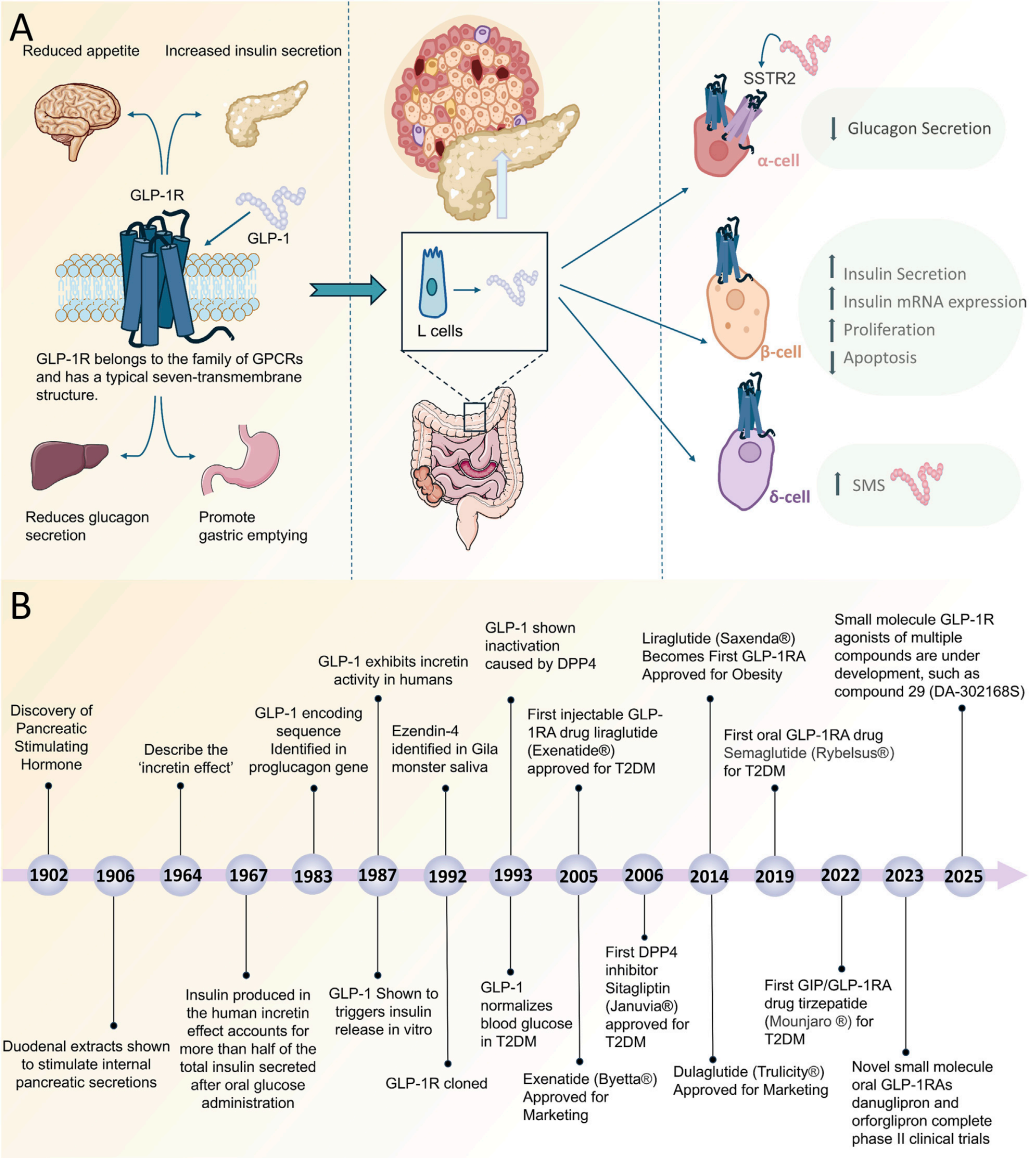
**(A)** Simplified Schematic of GLP-1R on α-cells, β-cells and δ-cells to lower blood glucose. **(B)** Timeline of discovery and clinical progression of GLP-1R agonists. Abbreviations: GPCRs, G protein-coupled receptors; EEC, enteroendocrine cells; GLP-1R, Glucagon-Like Peptide-1 Receptor; GLP-1RA, Glucagon-Like Peptide-1 Receptor agonists; SMS, somatostatin; SSTR2, somatostatin receptor type 2.

GLP-1R is a G protein-coupled receptor (Wu et al., 2020) (Figure 1A). Structurally, it contains an extracellular N-terminal domain (NTD) and a transmembrane domain (TMD) with α-helix bundles (Lei et al., 2018; Wu et al., 2020). The NTD of GLP-1R is one of its key regions for ligand binding. The C-terminus of the ligand first binds to the NTD, a process that is essential for the initial recognition of the ligand. And the TMD is responsible for further interaction with the N-terminus of the ligand, which triggers receptor activation (Graaf et al., 2016). Different ligands may trigger different conformational changes and activate different downstream signals. For example, some GLP-1R agonists primarily activate the insulin secretion signaling pathway, whereas others focus more on promoting insulin sensitivity or inhibiting glucagon secretion (Deganutti et al., 2022). Development of drugs characterized by biased activation may enable selective therapy and reduce adverse effects. Structural changes in the NTD also affect the ability of the ligand to bind to the receptor, which in turn affects receptor activation and drug efficacy (Ma et al., 2020).

GLP-1R is found mainly on the cell membranes of different cell types in the human body. In addition to α, β and δ cells in the pancreas, the receptor has been found in a variety of other tissues, including the intestine, stomach, lungs, heart, kidneys and several regions of the central nervous system (Holst et al., 2019; Abiola et al., 2024). GLP-1R is a specific receptor for glucagon-like peptide-1 (GLP-1) and GLP-1 acts by binding and activating GLP-1R (Müller et al., 2019). GLP-1 is an endocrine hormone secreted by enteroendocrine L-cells located in the distal jejunum, ileum and colon that express the glucagon gene and produce proglucagon (Lee et al., 1990). Proglucagon is stored in intracellular granules where it undergoes post-translational processing and is cleaved by prohormone convertase 1/3 to produce peptides such as GLP-1 (Orskov et al., 1986). GLP-1 is the main agonist of GLP-1R *in vivo*, while the main agonists *in vitro* are liraglutide, exenatide and Exendin-4. These receptor agonists target the GLP-1R and enter the body to treat a variety of diseases.

Subsequent studies have further revealed multiple physiological roles for GLP-1R, including its key role in glycemic control. GLP-1R Lowers blood glucose by binding to GLP-1, mainly by stimulating insulin secretion and inhibiting glucagon secretion (Drucker, 2018). GLP-1R binds to GLP-1 to activate multiple intracellular signaling pathways, including the Cyclic Adenosine Monophosphate/Protein Kinase A (cAMP/PKA) signaling pathway, the phosphatidylinositol 3-kinase/protein kinase B (PI3K/Akt) signaling pathway, and the pathways associated with anti-inflammatory and antioxidant stress responses (for details, refer to Chapter III). In recent years, the mechanism of action and therapeutic applications of GLP-1R have received extensive attention, especially in the field of diabetes and obesity treatment (Zheng et al., 2024). Studies have shown that GLP-1R not only plays a role in regulating blood glucose, but is also involved in a variety of metabolic and non-metabolic processes, including inhibition of gastric emptying, appetite control, and improvement of cardiovascular function (Cantini et al., 2016). In the future, with the in-depth exploration of the mechanism of GLP-1R action and the development of multi-target agonists (e.g., GLP-1R/GIPR dual agonists), GLP-1R-related therapeutics are expected to make breakthroughs in a wider range of disease areas (e.g., nonalcoholic steatohepatitis, AD, etc.), providing a more precise and highly effective strategy for the treatment of metabolic diseases and related complications.

## 3 Mechanism of action of GLP-1R in lowering blood glucose

GLP-1R is expressed on pancreatic α-cells, β-cells and δ-cells and reduces blood glucose levels directly or indirectly (Figure 1A). The main mechanism is to act on pancreatic β-cells to stimulate their insulin secretion. The GLP-1R is activated by binding to its ligand GLP-1. When GLP-1R on β-cells is activated, it binds to G proteins, particularly Gαs proteins, which increases the intracellular concentration of cAMP (Baggio and Drucker, 2007; Campbell and Drucker, 2013; Sandoval and D’Alessio, 2015). This increased cAMP activates PKA which in turn promotes insulin synthesis and secretion and inhibits glucagon release (Yang and Yang, 2016). Insulin gene expression is upregulated in pancreatic β-cells after GLP-1R activation, which increases insulin mRNA expression through activation of transcription factors such as pancreatic and duodenal homeobox-1 and cAMP-response element binding protein (Habener and Stoffers, 1998). GLP-1R activation promotes β-cell proliferation and anti-apoptosis through the PI3K/Akt pathway (Table 1), contributing to an increase in β-cell numbers (Liu et al., 2024). Furthermore, GLP-1 also activates Rap-induced insulin secretion via exchange protein directly activated by cAMP (Tengholm and Gylfe, 2017; Shahwar et al., 2024). In conclusion, GLP-1R activation triggers a series of complex physiological responses in pancreatic β-cells that work together to increase insulin secretion, inhibit β-cell apoptosis and stimulate proliferation, protect β-cells and ultimately regulate blood glucose.

**TABLE 1:** Mechanism of glucose-lowering action of GLP-1R.

Signaling pathways	Mechanism of action	Results	References
cAMP/PKA	Upon activation, GLP-1R binds to G proteins, leading to increased intracellular cAMP levels. cAMP activates PKA, which in turn promotes insulin synthesis and secretion and inhibits glucagon release	Increase insulin secretion, inhibit glucagon secretion, lower blood glucose	Huang et al. (2022), Chen et al. (2023)
PI3K/Akt	GLP-1R activation promotes β-cell proliferation and anti-apoptosis through the PI3K/Akt signaling pathway, increasing the number of β-cells	Protects β-cells, increases β-cell number, enhances insulin secretion	Liu et al. (2024)
Rap1	GLP-1 activation promotes Rap1-mediated insulin secretion via direct cAMP-activated exchange protein	Increases insulin secretion	Shahwar et al. (2024)
Calcium	GLP-1 reduces glucagon secretion by inhibiting P/Q-type Ca^2+^ channels on alpha cell membranes through activation of GLP-1R	Inhibits glucagon secretion and lowers blood glucose	Zheng et al. (2024)
MAPK	GLP-1R activates the MAPK pathway and promotes the proliferation and differentiation of pancreatic β-cells, thereby enhancing insulin synthesis and secretion	Increased insulin synthesis and secretion	Ma et al. (2018)
NF-κB	Inhibition of NF-κB activation by GLP-1R reduces macrophage infiltration and inhibits pro-inflammatory cytokine expression to enhance insulin sensitivity	Increased insulin sensitivity	Ma et al. (2018), Li et al. (2020)
Nrf2	GLP-1R improves pancreatic β-cell function and attenuates diabetic complications by activating the Nrf2 signaling pathway	Protecting pancreatic beta cells	Wang et al. (2024)
Wnt/β-catenin	GLP-1R promotes proliferation and enhanced function of pancreatic β-cells through activation of the Wnt/β-catenin signaling pathway, improving insulin secretion and glycemic control	Increased insulin secretion and decreased glycemic control	Kang et al. (2023)

Interestingly, only a very small amount of GLP-1R is present in the α-cell membrane, but GLP-1 can inhibit glucagon secretion in human pancreatic islets by directly acting on GLP-1R on α-cells. The mechanism is that GLP-1 exerts its glucagon inhibitory effect in human pancreatic islets through an intrinsic (non-paracrine) PKA-dependent effect mediated by the activation of the few GLP-1Rs present in the plasma membrane of α-cells, which ultimately inhibits P/Q-type Ca2+ channels (Ramracheya et al., 2018). Glucagon, secreted by alpha cells in the pancreas, is an important hormone that promotes the release of glucose from the liver into the bloodstream (Robertson, 2023). Therefore, it can be concluded that inhibiting glucagon secretion is critical in situations where blood glucose levels need to be controlled or lowered. It has been shown that glucagon release can be inhibited by activating the mitogen-activated protein kinase pathway, enhancing the transcription of Paired Box 6 and Proglucagon genes, and further promoting the production of GLP-1 and activation of GLP-1R in α-cells (De Marinis et al., 2010). Evidence for this action has been demonstrated in mice (Fridolf et al., 1991), rats (de Heer et al., 2008), dogs (Freyse et al., 1997), and humans *in vivo* (Hare et al., 2009), as well as in intact isolated mouse islets (De Marinis et al., 2010). In addition to direct actions, pancreatic β-cells can also be affected by paracrine actions that promote insulin production and release, thereby indirectly inhibiting glucagon secretion (Saltiel and Kahn, 2001; Campbell and Newgard, 2021). β-cell GLP-1R signaling activates α-cell GLP-1 production via paracrine signaling factors. GLP-1 inhibits glucagon release by regulating α-cell N- and L-type Ca2+ channel-dependent cytokinesis (De Marinis et al., 2010).

GLP-1R is present not only in α-cells and β-cells, but also in δ-cells. The increase in cAMP levels in δ-cells is a consequence of GLP-1R activation in these cells, which in turn enhances the secretion of growth inhibitory hormone through signaling pathways such as PKA and Calcium/Calmodulin-dependent protein kinase (Brereton et al., 2015). Growth inhibitory hormone, a widespread and potent inhibitory hormone, has been shown to inhibit the activity of a variety of endocrine cells, including glucagon secretion from pancreatic islet α-cells (Brereton et al., 2015). Furthermore, it has been demonstrated that this hormone can indirectly affect α-cells by inhibiting the secretion of other hormones (Strowski et al., 2000; Hauge-Evans et al., 2009; Brereton et al., 2015). In their subsequent research, Hauge-Evans et al. investigated the role of δ-cell growth inhibitors in insulin and glucagon secretion, utilizing the Sst−/− mouse model (Hauge-Evans et al., 2009). Their findings revealed that Sst−/− mice exhibited augmented insulin and glucagon secretory responses in comparison with the control group (Hauge-Evans et al., 2009). Moreover, this enhanced secretion could be suppressed by exogenous growth inhibitors. These results suggest that the growth inhibitor of the delta cells exerts a sustained inhibitory effect on the secretion of insulin and glucagon, and that it may promote the responses of the islets to activation by cholinergic stimuli. In summary, GLP-1R on δ-cells is activated and promotes growth inhibitory hormone secretion, creating a multilayered inhibitory mechanism that effectively reduces glucagon secretion, and in turn lowers blood glucose levels.

It has recently been shown that GLP-1R neurons in the dorsomedial hypothalamic nucleus regulate pancreatic activity by affecting the dorsal motor nucleus of the vagus, specifically lowering blood glucose levels by increasing insulin release (Huang et al., 2022). In addition, GLP-1R reduces gluconeogenesis by delaying gastric emptying and decreasing appetite, which also contributes to glycemic control (Xie et al., 2022). In a word, GLP-1R can exert glucose-lowering effects through multiple mechanisms.

## 4 GLP-1R clinical trial progress

### 4.1 GLP-1R agonists in the treatment of DM

GLP-1R agonists have emerged as a significant therapeutic agent in the management of diabetes. These drugs mimic the physiological effects of GLP-1 by binding to GLP-1R, which enhances insulin secretion, inhibits glucagon release, delays gastric emptying, and reduces food intake (Drucker, 2018). Consequently, this results in a reduction of blood glucose levels and a decrease in body weight. In 1902, Bayliss and Starling made the first discovery of a hormone capable of stimulating pancreatic secretion, glucagon (Bayliss and Starling, 1902) (Figure 1B). Subsequently, Moore proposed the hypothesis that intestinal secretions might have a glycemic-lowering effect in diabetics, which was the first study to suggest an effect of intestinal secretions on blood glucose (Moore, 1906), and in the 1960s a team of researchers in the UK and the US independently discovered the “incretin effect,” in which oral glucose markedly stimulates insulin secretion, setting the stage for the subsequent development of incretin (Elrick et al., 1964; McIntyre et al., 1964). In 1967, Perley and Kipnis conducted a further study in which they showed that the insulin produced during the incretin effect in humans accounted for more than half of the total insulin secreted by oral glucose ingestion (Perley and Kipnis, 1967). In 1983, Bell et al. confirmed that GLP-1 was cleaved from proglucagon in the gut by cloning and sequencing the mammalian glucagon gene (Bell et al., 1983a; Bell et al., 1983b). This discovery was a major step forward in the study of GLP-1R agonists.

The 21st century has seen significant progress in GLP-1R agonist research, with liraglutide (exenatide) becoming the first GLP-1R agonist to be approved by the US Food and Drug Administration (FDA) for the treatment of T2DM in 2005 (Jensterle et al., 2022). Since then, other GLP-1R agonists such as exenatide (Byetta^®^) and dulaglutide (Trulicity^®^) have been approve (Holman et al., 2017; Gerstein et al., 2019). Semaglutide oral tablets (Rybelsus^®^), with 94% homology to human GLP-1, were approved in 2019 as the first GLP-1R agonists for oral administration (Andersen et al., 2021). Although oral Semaglutide offers a non-injectable dosing option, it faces a number of challenges, notably bioavailability, metabolic stability, and gastrointestinal side effects (Ke et al., 2024) (Table 2). Second-generation oral semaglutide, whose bioavailability and safety have been significantly improved by the application of new excipients and formulation optimization, but its gastrointestinal side effects are still obvious (Nielsen et al., 2025). Therefore, the development of an oral delivery strategy for peptide GLP-1R agonists is expected to reduce these adverse effects and improve patient compliance and quality of life (Borner et al., 2020; Borner et al., 2022). Meanwhile, multi-target agonists are of increasing interest to researchers. Coskun et al. reported LY3298176, a novel dual GIP and GLP-1R agonist for the treatment of type 2 diabetes, a study that demonstrated its potential from discovery to clinical proof-of-concept (Coskun et al., 2018). Rosenstock et al. published the results of a phase 3 clinical trial of tirzepatide, a dual GIP and GLP-1 receptor agonist, in patients with T2DM, showing significant results in glycemic control and weight loss (Rosenstock et al., 2021). In a rodent model, a novel GLP-1R/GIPR/GCGR triple agonist, SAR441255, has been shown to promote weight loss and the maintenance of healthy blood glucose levels and is superior to the GLP-1R/GCGR dual agonist (Bossart et al., 2022). In 2023, Juan P Frias and colleagues, in The Lancet, published the first phase two clinical trial of orforglipron, which investigated the use of a small-molecule oral GLP-1R agonist called orforglipron in type 2 diabetes, which can be taken at any time of the day without restrictions on food and water intake (Frias et al., 2023). In the same year, danuglipron, another small molecule oral GLP-1R agonist, also underwent phase two clinical trials. The study recruited 411 patients and showed that danuglipron reduced glycated hemoglobin, fasting blood glucose and body weight at week 16 compared with placebo and was well tolerated without the need for injections or fasting restrictions (Saxena et al., 2023). However, due to danuglipron’s potential for drug-induced liver damage, 2025 Pfizer has announced that it is discontinuing development (Pfizer, 2025). Currently, another small molecule GLP-1R agonist called compound 29 (DA-302168S) has also shown good therapeutic potential (Dong et al., 2025). This compound has shown increased activity both *in vitro* and *in vivo* with a reduced risk of drug-drug interactions and is undergoing phase 2 clinical trials.

**TABLE 2:** Overview of GLP-1R agonist efficacy and safety in major clinical trials.

Test name	Name of drug	Year	Effectiveness	Type of adverse reaction	Clinical benefit beyond DM control	References
DURATION-1	Exenatide	2006	Weekly exenatide outperformed twice-daily exenatide in lowering glycated hemoglobin (HbA1c) reduction, with more patients hitting the HbA1c ≤ 7.0% target at 30 weeks	Nausea (26.4%), vomiting (18.6%), injection site itching (17.6%)	Reduced total cholesterol and VLDL-C levels, with potential cardiovascular protection benefits	Drucker et al. (2008)
LEAD-6	Liraglutide	2007	Daily liraglutide was significantly superior to exenatide twice daily in lowering HbA1c (1.12% vs. 0.79% reduction in HbA1c)	Gastrointestinal adverse reactions, mild hypoglycemic events (26%), headache (8.9%), back pain (6.0%), upper respiratory tract infection (6.4%)	Reduces body weight, improves blood lipids, and is more effective than exenatide in lowering triglycerides and free fatty acids	Buse et al. (2009)
T-EMERGE-2	Taspoglutide	2008	Taspoglutide monotherapy significantly reduced HbA1c and fasting blood glucose (FPG) in type 2 diabetes patients who had not received prior glucose-lowering medication, after 24 weeks of treatment	Nausea and vomiting, hypersensitivity reactions occurred in 4 patients	Weight loss and significant improvement in beta cell function	Raz et al. (2012)
GETGOAL-X	Lixisenatide	2008	Lixinatide once daily was comparable to exenatide twice daily in lowering HbA1c, and both were similar in lowering FPG, but exenatide was slightly better in reducing body weight	Adverse gastrointestinal reactions, with 8.5% of patients reporting injection site reactions	Reduced risk of hypoglycemia, good performance in terms of gastrointestinal tolerance	Rosenstock et al. (2021)
ELIXA	Lixisenatide	2010	Lixisenatide significantly reduces T2DM in combined acute coronary syndrome patients, especially those with pre-existing high albuminuria at baseline	Gastrointestinal adverse effects, doubling of serum creatinine	Nephroprotective effect	Muskiet et al. (2018)
AWARD-1	Dulaglutide	2010	Once-weekly Dulaglutide (1.5 mg and 0.75 mg) significantly reduced HbA1c, superior to placebo and exenatide, and resulted in higher rates of glycemic compliance at weeks 26 and 52	Gastrointestinal adverse effects, hypoglycemia, 1 patient died of myocardial infarction	Weight loss	Wysham et al. (2014)
REWIND	Dulaglutide	2011	Once-weekly treatment with Dulaglutide significantly reduced the risk of cardiovascular events, including nonfatal myocardial infarction, stroke, and cardiovascular death, in patients with type 2 diabetes by 12% compared with placebo	2.4% for serious gastrointestinal events, 0.5% for serious hepatic events, 1.7% for serious renal or urinary events, 4.4% for cardiac arrhythmias or cardiovascular conduction disturbances, and 1.3% for serious hypoglycemia. 47.4% of participants reported adverse gastrointestinal events	Reduced microvascular complications (including diabetic retinopathy and nephropathy), lower body weight, systolic blood pressure and VLDL-C	Gerstein et al. (2019)
SUSTAIN-3	Semaglutide	2013	Once-weekly Semaglutide 1.0 mg was superior to once-weekly exenatide extended-release dosage form 2.0 mg in lowering HbA1c and reducing body weight	Gastrointestinal adverse reactions, lipase increased, nasopharyngitis, headache, and severe hypoglycemic events were reported in 8.2% of subjects	Significantly lowered systolic blood pressure and improved lipid profiles (e.g., free fatty acids, VLDL-C, and triglycerides)	Ahmann et al. (2018)
PIONEER-4	Semaglutide	2016	Oral Semaglutide is significantly better than placebo in reducing HbA1c and body weight, and was superior to subcutaneous liraglutide at both 26 and 52 weeks of treatment	Gastrointestinal adverse reactions, nasopharyngitis, headache loss of appetite, back pain, and 8% of participants reported serious adverse reactions	Improved patient compliance as an oral medication	Pratley et al. (2019)
SURPASS-1	Tirzepatide	2019	All dose groups significantly reduced HbA1c levels, with 87%–92% of patients having HbA1c <7.0% and 31%–52% having HbA1c <5.7% after 40 weeks of treatment without an increased risk of severe hypoglycemia	Gastrointestinal adverse reactions, injection site reactions 2%–3%, hypersensitivity reactions 1%–2%, loss of appetite 5%–8%, headache 4%–5%, and constipation 6%–8%	Weight loss with improvements in lipids, insulin sensitivity and systolic blood pressure	Rosenstock et al. (2021)
GLORY-1	Retatrutide	2021	Retatrutide achieved substantial weight loss in obesity treatment. By 48 weeks, those on the 12 mg dose lost an average of 24.2% of their body weight, and all on 8 mg or higher doses lost over 5%, showing a robust dose-dependent effect	Gastrointestinal adverse effects, Skin sensation abnormalities, transient increases in alanine aminotransferase in a few participants	Improved several cardiovascular metabolic markers, including blood pressure, glycosylated hemoglobin, fasting glucose, insulin and lipid levels (except HDL cholesterol)	Jastreboff et al. (2023)
TRIUMPH-1	Retatrutide	2023	In clinical trials			Lilly (2023)
ATTAIN-HYPERTENSION	Orforglipron	2025	In clinical trials			Lilly (2025)

### 4.2 Others

Cardiometabolic diseases revolve around the axis of insulin resistance and deficiency. Recent studies have reported that the development of heart failure in adolescents is closely associated with a dramatic increase in DM, obesity, and metabolic dysfunction-associated steatotic liver disease (MASLD) (Wu et al., 2024; Parizad et al., 2025). In addition, recent trends in the burden of vascular risk factors in young adult ischemic stroke patients have attracted attention (Shoskes et al., 2025). In this context, GLP-1R agonists show remarkable potential as an emerging therapeutic agent.GLP-1R agonists reduce body weight, lower blood pressure, and improve lipid levels, as well as exerting anti-inflammatory and antioxidant effects by directly acting on GLP-1R in the heart and blood vessels, thereby reducing the risk of atherosclerotic thrombotic events (Solini et al., 2023). Studies have shown that GLP-1R agonists are also able to reduce myocardial infarction, stroke and heart failure (Solini et al., 2023; Taal and Selby, 2025). Furthermore, the role of GLP-1R agonists in the treatment of neurodegenerative diseases such as Alzheimer’s disease (AD) and Parkinson’s disease (PD) is being actively explored (Siddeeque et al., 2024). GLP-1R agonist can slow down the AD process by reducing Aβ deposition and inhibiting tau protein hyperphosphorylation (Jantrapirom et al., 2020; Kang et al., 2023). And it can cross the blood-rain barrier, reduce inflammation and oxidative stress, and protect synapses and neurons, showing neuroprotective effects in animal models of AD and PD (Hong et al., 2024). In addition, GLP-1R agonist have shown promise in the MASLD (Romero-Gómez et al., 2023). GLP-1R agonists have an important role in reducing liver fat and fibrosis in patients with MASLD. Recent studies have shown that GLP-1R agonists can significantly improve hepatic fat content and liver function indexes in patients with MASLD, thereby reducing the risk of progression to Chronic Liver Disease in patients with MASLD (Singh et al., 2024; Soresi and Giannitrapani, 2024).

Despite the great therapeutic potential of GLP-1R agonists, some challenges and shortcomings remain. For example, the mechanism of action of GLP-1R in non-islet function is not fully understood and further studies are needed to elucidate it. In addition, the long-term effects and side effects of GLP-1R agonists need more in-depth studies. Nevertheless, the research and development of GLP-1R agonists provide new perspectives and strategies for the treatment of diabetes, obesity, and related complications.

## 5 Conclusion

DM as a major challenge in global public health, is showing a serious trend of increasing prevalence year by year. This metabolic disease involves defects in insulin secretion and action, and is closely linked to obesity, genetic factors, and metabolic inflammation, and its complex pathologic mechanisms are driving the deepening of cutting-edge research in endocrine medicine. In recent years, GLP-1R and its agonists have become a hot research topic and a star drug in the field of diabetes treatment. GLP-1R triggers various intracellular signaling pathways, such as the cAMP/PKA and PI3K/Akt pathways, by binding to endogenous GLP-1 or exogenous agonists, resulting in the enhancement of insulin secretion, inhibition of glucagon secretion, as well as anti-inflammatory and antioxidant stress response.

Since the first GLP-1R agonist, exenatide, was approved for the treatment of T2DM in 2005, a variety of GLP-1R agonists such as liraglutide and dulaglutide have been approved. However, GLP-1R agonists also have many challenges and limitations. The complex structure of GLP-1R poses a challenge for drug development. Researchers should study its binding sites and conformational changes in depth to provide theoretical support for the design of highly selective drugs. GLP-1R agonists typically have short half-lives and require frequent dosing. Chemical modifications, such as amino acid mutations, addition of fatty acid chains, or design of synthetic analogs, can enhance their resistance to enzymatic degradation and significantly improve metabolic stability and half-life (Yu et al., 2018; Wan et al., 2023). In addition, the limitations of GLP-1R agonist therapy, which may cause gastrointestinal adverse effects and significantly alter the patient’s physiological state, make it not widely applicable to all patients. Although it is challenging to develop individualized therapeutic regimens, personalized medicine, such as genetics and biomarker testing, can be used to tailor therapies to patients’ specific conditions, thereby improving the safety and efficacy of treatments (Sugandh et al., 2023). With advances in genetic testing technology, it is possible to more accurately diagnose monogenic diabetes and provide individualized treatment for non-obese diabetic patients (Murphy et al., 2023). The development of novel drug delivery systems or formulations is also essential to improve patient compliance and reduce gastrointestinal adverse effects. Examples include small molecule GLP-1R agonists, multiple agonists, and salcaprozate sodium (SNAC) (Twarog et al., 2019; Zhang et al., 2024; Anderer, 2025). In addition, more extensive long-term clinical trials and post-marketing surveillance are to be conducted to obtain better long-term safety data. Future studies should focus on exploring the multi-target synergistic effects of GLP-1R. Developing and optimizing multi-target agonists could potentially address multiple aspects of metabolic disorders simultaneously. For example, GLP-1R/GIPR dual agonists have demonstrated superior glucose-lowering and weight-loss effects in the treatment of diabetes and obesity. In addition, the activation of GLP-1R is to be explored in other diseases such as Alzheimer’s disease, Parkinson’s disease, non-alcoholic fatty liver disease and non-alcoholic steatohepatitis in addition to traditional diabetes treatment. In conclusion, the activation and promising application of GLP-1R offer broad possibilities for the future treatment of diabetes and related diseases. With the in-depth study of the structure and function of GLP-1R and the development of novel agonists, it is expected to become a more efficient and safer therapeutic option.
